# Wild food plants of Brazil: a theoretical approach to non-random selection

**DOI:** 10.1186/s13002-023-00603-6

**Published:** 2023-07-08

**Authors:** Lailson César Andrade Gomes, Patrícia Muniz de Medeiros, Ana Paula do Nascimento Prata

**Affiliations:** Campus of Engineering and Agricultural Sciences of the Federal University of Alagoas, Rio Largo, Brazil

**Keywords:** Unconventional food plants, Wild edible plants, Theory-based ethnobotany, Plants native to Brazil

## Abstract

Ethnobiological investigations have focused on identifying factors that interfere with the criteria adopted for selection of plants, especially medicinal plants, by different populations, confirming the theory that plant selection is not random. However, regarding wild food plants, little effort has been made to confirm the theory in this context, especially in Brazil. Therefore, this systematic review aimed to contribute to the establishment of theoretical bases of the non-random selection of wild food plants by local populations in Brazil. For this, searches were made in 4 databases, namely, Web of Science, Scielo, Scopus and PubMed, using 8 sets of keywords in English and Portuguese in order to identify wild food plants occurring in Brazil. The steps were: application of inclusion and exclusion criteria, screening of articles, selection of studies based on risk of bias, data treatment and, finally, data analysis. Eighty articles met the inclusion criteria of this review. However, 45 of them were considered to present high risk of bias and thus 35 articles were kept for the identification of overused and underused families. The results were inferred through two different approaches (IDM and Bayesian). Annonaceae, Arecaceae, Basellaceae, Cactaceae, Capparaceae, Caryocaraceae, Myrtaceae, Passifloraceae, Rhamnaceae, Rosaceae, Sapotaceae, Talinaceae, and Typhaceae were considered overused. Eriocaulaceae, Orchidaceae, and Poaceae were considered underused. Therefore, considering that some families are more (or less) used than others, we confirm that the wild food plants occurring in Brazil, known and used by different populations, are not chosen at random.

## Introduction

Ethnobiological investigations around the world have focused on identifying the criteria to select plants, especially those used in medicinal applications, in different populations. Among the different factors that can interfere with plant selection, taxonomic and phylogenetic aspects are addressed in a large number of studies, which are based on the theory of non-random selection, which states that plants can be overused or underused depending on factors that will determine their selection or not. One of the pioneering studies in this regard [1] investigated whether the use of medicinal plants by Native Americans was effective or placebo medicine only. Using a regression analysis, the author came to the conclusion that some taxonomic groups were more used than what was expected if plants were being randomly selected. Years later, seeking to understand the motivations for selectivity, Moerman [2] reported that the presence of biologically active properties as well as factors related to the knowledge about plants acquired over the years and passed from generation to generation contributed to the selection of some plants.

More recently, ethnobiological studies using different approaches and statistical tools have confirmed the theory that plants are not selected at random, but there are rather taxonomic biases that determine why some species are preferred over others [3–8]. There are other approaches using phylogenetic tools which also confirm this theory. These studies consider that closer species share characteristics that justify their use, and for this reason, there are groups that stand out, indicating that species of those groups are selected precisely because they have favorable characteristics, leading to the rejection of the possibility of randomness [9–13].

Some of the plant selection criteria, which can culminate in taxonomic biases, have been found and they are associated with availability, historical and cultural preferences, presence of alkaloids, terpenoids, and biologically active volatile compounds in the case of medicinal plants [6]. However, regarding wild food plants, little effort has been made to test the theory of non-random selection of plants, especially in Brazil, as there are no reports of investigations with this scope.

Among the multiple tools used to test the theory of non-random plant selection, two approaches are frequent in different socio-ecological contexts to demonstrate which taxonomic groups are the most used. The first is the Bayesian model, which assumes uncertainty only on the number of species of the investigated flora, that is, the number of useful plants [14]. The other is the Imprecise Dirichlet Model (IDM), which assumes that both the data on the number of species of the investigated flora and the data of the number of species of the overall flora of the investigated environment are uncertain [15]. The first studies in this sense used residual analysis of simple linear regressions to show overused and underused families [1], but this proposal was questioned due to the statistical inconsistency of the method [16]. Then, a binomial analysis was proposed [16], but it was also objected [14].

In this review, we aim to contribute to the establishment of theoretical bases for the theory of non-random plant selection by local populations, specifically in the context of food plants of the Brazilian flora, using the approaches of IDM and Bayesian model to identify patterns in the knowledge and use of wild food plants in Brazil from the identification of over- and underused families. The following question was the starting point: Are there botanical families over- or underused for food purposes by local populations in Brazil? Our hypothesis is that some botanical families are overused and others underused.

## Methodology

### Bibliographic search

We searched for scientific documents with an ethnobotanical approach that presented a list of food plants occurring in Brazil with at least one species. To this end, four databases were consulted: Web of Science, Scielo, Scopus and PubMed. Search queries were run using pre-established keywords, namely: (1) "Unconventional Food Plants" AND Brazil; (2) "Wild Food Plants" AND Brazil; (3) "Wild Edible Plants" AND Brazil; (4) “Useful Plants” AND Ethnobotany AND Brazil; (5) "Plantas Comestíveis" AND Brasil; (6) "Plantas Alimentícias Não Convencionais" AND Brasil; (7) "Plantas Alimentícias Silvestres" AND Brasil; (8) “Plantas Úteis” AND Etnobotânica AND Brasil. Search results refer to the knowledge and/or use of food plants. Searches were performed on the title, abstract and keywords of the articles.

### Inclusion/exclusion criteria

Only studies published in Portuguese and English were included in the review. Works with more general approaches (useful plants) were selected for later extraction of data regarding food plants. Review articles were excluded, but their references were used for locating further articles with primary data. Studies conducted in the same community or using the same database were excluded and the one that contained more complete and detailed information was included. Also, studies that used systematic instruments for data collection, such as interviews, were included. We excluded studies that did not provide information about the data collection method and also those that did not mention the scientific names of the species.

### Screening

Duplicates, that is, articles found more than once in different databases, were excluded; only one document was entered in the database. Subsequently, the abstract of each article was read and those without an ethnobotanical approach and reviews were removed (reviews were used for another purpose as mentioned in the inclusion/exclusion criteria section). Then, a second screening was performed. The articles selected in the first screening were read in full length. Those that did not present a list of species and those that did not identify the species were excluded.

### Study selection method based on risk of bias

After application of inclusion/exclusion criteria and screening steps, the articles were classified as presenting low, moderate, and high risk of bias according to criteria for ethnobotanical studies of medicinal plants based on sample quality [17].

Articles presenting moderate and low risk underwent another classification that informed a possible increase in the level of risk based on the following information: complete or incomplete identification of plant material; presentation of a complete or partial list of species; presence of restrictions in the studied habit or taxonomic groups, for example, studies conducted only with herbs or forest species or studies with only one family [18].

Finally, articles classified as presenting moderate and low risk were included in the analysis and the others were removed.

### Treatment of data

Data on food species and place where the study was carried out were extracted from each article according to the following information: bibliographic reference, biome, region, state, scientific name, family, popular name, part used, and form of use.

Information on all species occurring in Brazil was further extracted using the flora package in R [19]. The information included: scientific name, family, life form, habitat, type of vegetation, and establishment (origin) according to the listing of Flora do Brasil [20]. The correct spelling and accepted names of the species were checked also using this database. When a species was not mentioned in the listing of Flora do Brasil, the database World Flora Online was consulted [21].

Only the list of accepted native Angiosperm species was extracted from the listings of Flora do Brasil [20] and World Flora Online [21]. Naturalized, exotic, cultivated species, and those without the source information were excluded.

### Data analysis

Two distinct approaches were used to identify overused and underused families: the Bayesian model based on Weckerle et al. [14] and the IDM based on Weckerle et al. [15]. While the Bayesian model assumes uncertainty only in the number of native food species, the IDM assumes that data on both the number of native food species and the number of overall native species are uncertain. The Excel Inv.BETA function was used calculate the range of the most probable values of θ (proportion of native food species for the overall flora) and θj (proportion of native food species for family j).

Families which obtained a lower limit of θj greater than the upper limit of θ were considered to be overused. Families which obtained a upper limit of θj lower than the lower limit of θ were considered underused. In cases of overlap between the limits of θj and θ, the family was considered neither over- nor underused.

## Results

Eighty articles met the inclusion criteria. However, 45 of them were considered to present a high risk of bias, 17 a moderate risk, and 18 a low risk, according to the categorization of risks of bias in ethnobotanical studies in Brazil [17, 18]. Table 1 lists the 35 articles that composed this review.

**Table 1:** Listing and general aspects of studies with an ethnobotanical approach addressing wild food plants carried out in Brazil

References	State	Region	Ecosystem	Community type	Area
Albuquerque et al. [22]	Pernambuco	NO	CE	Rural	R
Alves et al. [23]	Paraíba	NO	CA	Rural	R
Baptista et al. [24]	Rio Grande do Sul	S	AF	Artisanal fishermen	U
Barreira et al. [25]	Minas Gerais	SE	AF	Rural	R
Borges and Peixoto [26]	Rio de Janeiro	SE	AF	Caiçaras^1^	R
Bortolotto et al. [27]	Mato Grosso do Sul	MW	PAN	Rural	R
Brito and Senna-Valle [28]	Rio de Janeiro	SE	AF	Caiçaras^1^	N/i
Campos et al. [29]	Ceará	NO	CA	Extractivists	R
Chaves et al. [30]	Piauí	NO	CA	Rural	R
Christo et al. [31]	Rio de Janeiro	SE	AF	Rural	R
Conde et al. [32]	Minas Gerais	SE	AF	Quilombola^2^	R
Crepaldi and Peixoto [33]	Espírito Santo	SE	AF	Quilombola^2^	R
Florentino et al. [34]	Pernambuco	NO	CA	N/i	N/i
Fonseca-Kruel and Peixoto [35]	Rio de Janeiro	SE	AF	Artisanal fishermen	U
Gandolfo and Hanazaki [36]	Santa Catarina	S	AF	Native	R
Hanazaki et al. [37]	São Paulo	SE	AF	Caiçaras^1^	R
Leal et al. [38]	Santa Catarina	S	AF	Rural	U
Lobo et al. [39]	Pernambuco	NO	AF	Gypsies	N/i
Lopes and Lobão [40]	Espírito Santo	SE	AF	Artisanal fishermen	R
Lucena et al. [41]	Paraíba	NO	CA	Rural	R
Lucena et al. [42]	Paraíba	NO	CA	Rural	R
Medeiros et al. [43]	Alagoas	NO	AF	Farmers	R
Medeiros et al. [44]	Bahia	NO	CA	Rural	R
Moura et al. [45]	Sergipe	NO	AF	Artisanal fishermen	R
Nascimento et al. [46]	Pernambuco	NO	CA	Rural	R
Nascimento et al. [47]	Pernambuco	NO	CA	Rural	R
Nunes et al. [48]	Paraíba	NO	CA	Rural	R
Pedrosa et al. [49]	Paraíba	NO	CA	Rural	R
Ribeiro et al. [50]	Paraíba	NO	CA	Rural	R
Rodrigues et al. [51]	São Paulo	SE	AF	Quilombola^2^	R
Roque and Loiola [52]	Rio Grande do Norte	NO	CA	Rural	R
Santos et al. [53]	Sergipe	NO	AF	Farmers	R
Santos et al. [54]	Ceará and Pernambuco	NO	CA	Rural	R
Strachulski and Floriani [55]	Paraná	S	AF	Rural	R
Tuler et al. [56]	Minas Gerais	SE	AF	Farmers	R

Region: *S* South, *SE* Southeast, *MW* Midwest, *NO* Northeast, *N* North. Ecosystem: *AF* Atlantic Forest, *PAN* Pantanal, *CA* Caatinga. Area: *U* Urban, *R* Rural, *N/i* no information

^1^Traditional inhabitants of the coast of southeastern Brazil; ^2^ Descendants of Afro-Brazilian runaway slaves living in hideouts up-country called Quilombos

The overused and underused families are listed in Table 2. The Bayesian approach indicated 14 overused and 3 underused families. The IDM was more conservative, indicating a total of 13 overused families and only 1 underused family.

**Table 2:** Overused and underused families of wild food plants from the Brazilian flora

Family (*J*)	nj	xj	Lower (*B*)	Upper (*B*)	Status (*B*)	Lower (*I*)	Upper (*I*)	Status (*I*)
Acanthaceae	472	0	0.0000000	0.0077850	ns	0.0000000	0.0183457	ns
Achariaceae	19	0	0.0000000	0.1764669	ns	0.0000000	0.3491221	ns
Achatocarpaceae	1	0	0.0000000	0.9750000	ns	0.0000000	0.9936905	ns
Adoxaceae	2	0	0.0000000	0.8418861	ns	0.0000000	0.9472550	ns
Alismataceae	35	1	0.0007231	0.1491721	ns	0.0006660	0.2480494	ns
Alstroemeriacea	41	0	0.0000000	0.0860438	ns	0.0000000	0.1865620	ns
Amaranthaceae	132	0	0.0000000	0.0275592	ns	0.0000000	0.0635684	ns
Amaryllidacea	131	0	0.0000000	0.0277666	ns	0.0000000	0.0640326	ns
Anacardiaceae	58	5	0.0285860	0.1898260	Overused	0.0271514	0.2421587	Overused
Anisophylleaceae	3	0	0.0000000	0.7075982	ns	0.0000000	0.8818828	ns
Annonaceae	377	9	0.0109729	0.0448327	Overused	0.0108858	0.0545119	Overused
Apiaceae	70	1	0.0003616	0.0770438	ns	0.0003468	0.1343938	ns
Apocynaceae	787	4	0.0013865	0.0129619	ns	0.0013812	0.0181709	ns
Apodanthaceae	2	0	0.0000000	0.8418861	ns	0.0000000	0.9472550	ns
Aptandraceae	10	0	0.0000000	0.3084971	ns	0.0000000	0.5381315	ns
Aquifoliaceae	54	1	0.0004687	0.0989152	ns	0.0004441	0.1700398	ns
Araceae	504	0	0.0000000	0.0072925	ns	0.0000000	0.0171943	ns
Araliaceae	94	0	0.0000000	0.0384834	ns	0.0000000	0.0877318	ns
Arecaceae	300	27	0.0601495	0.1282425	Overused	0.0595455	0.1383209	Overused
Aristolochiacea	84	0	0.0000000	0.0429649	ns	0.0000000	0.0974808	ns
Asparagaceae	14	0	0.0000000	0.2316358	ns	0.0000000	0.4343179	ns
Asphodelaceae	1	0	0.0000000	0.9750000	ns	0.0000000	0.9936905	ns
Asteraceae Bercht	2066	9	0.0019938	0.0082533	ns	0.0019909	0.0101093	ns
Balanophoraceae	15	0	0.0000000	0.2180194	ns	0.0000000	0.4141775	ns
Basellaceae	2	1	0.0125791	0.9874209	Overused	0.0050508	0.9949492	ns
Bataceae	1	0	0.0000000	0.9750000	ns	0.0000000	0.9936905	ns
Begoniaceae	215	0	0.0000000	0.0170112	ns	0.0000000	0.0396879	ns
Berberidaceae	3	0	0.0000000	0.7075982	ns	0.0000000	0.8818828	ns
Bignoniaceae	411	1	0.0000616	0.0134812	ns	0.0000612	0.0245522	ns
Bixaceae	7	1	0.0036103	0.5787232	ns	0.0025286	0.7376219	ns
Bonnetiaceae	8	0	0.0000000	0.3694166	ns	0.0000000	0.6097426	ns
Boraginaceae	146	3	0.0042577	0.0588739	ns	0.0041716	0.0855848	ns
Brassicaceae	6	0	0.0000000	0.4592581	ns	0.0000000	0.7007049	ns
Bromeliaceae	1356	9	0.0030393	0.0125619	ns	0.0030326	0.0153734	ns
Brunelliaceae	1	0	0.0000000	0.9750000	ns	0.0000000	0.9936905	ns
Burmanniaceae	26	0	0.0000000	0.1322746	ns	0.0000000	0.2735152	ns
Burseraceae	117	2	0.0020769	0.0603860	ns	0.0020248	0.0945588	ns
Cabombaceae	5	0	0.0000000	0.5218238	ns	0.0000000	0.7551368	ns
Cactaceae	276	12	0.0226646	0.0747156	Overused	0.0224188	0.0871251	Overused
Calophyllaceae	94	0	0.0000000	0.0384834	ns	0.0000000	0.0877318	ns
Calyceraceae	6	0	0.0000000	0.4592581	ns	0.0000000	0.7007049	ns
Campanulaceae	57	0	0.0000000	0.0626675	ns	0.0000000	0.1392432	ns
Canellaceae	6	0	0.0000000	0.4592581	ns	0.0000000	0.7007049	ns
Cannabaceae	14	0	0.0000000	0.2316358	ns	0.0000000	0.4343179	ns
Cannaceae	4	0	0.0000000	0.6023646	ns	0.0000000	0.8159484	ns
Capparaceae	29	3	0.0218637	0.2735152	Overused	0.0197672	0.3643923	Overused
Caprifoliaceae	17	0	0.0000000	0.1950643	ns	0.0000000	0.3789268	ns
Cardiopteridaceae	5	0	0.0000000	0.5218238	ns	0.0000000	0.7551368	ns
Caricaceae	8	1	0.0031597	0.5265097	ns	0.0022990	0.6920953	ns
Caryocaraceae	16	2	0.0155136	0.3834762	Overused	0.0130122	0.5120293	Overused
Celastraceae	141	2	0.0017224	0.0502983	ns	0.0016865	0.0791681	ns
Ceratophyllaceae	2	0	0.0000000	0.8418861	ns	0.0000000	0.9472550	ns
Chloranthaceae	3	0	0.0000000	0.7075982	ns	0.0000000	0.8818828	ns
Chrysobalanaceae	280	2	0.0008662	0.0255628	ns	0.0008570	0.0407470	ns
Cistaceae	1	0	0.0000000	0.9750000	ns	0.0000000	0.9936905	ns
Cleomaceae	34	0	0.0000000	0.1028179	ns	0.0000000	0.2190962	ns
Clethraceae	2	0	0.0000000	0.8418861	ns	0.0000000	0.9472550	ns
Clusiaceae	140	2	0.0017348	0.0506509	ns	0.0016983	0.0797087	ns
Combretaceae	61	1	0.0004150	0.0879881	ns	0.0003955	0.1523635	ns
Commelinaceae	106	1	0.0002388	0.0514431	ns	0.0002322	0.0912983	ns
Connaraceae	71	0	0.0000000	0.0506294	ns	0.0000000	0.1139373	ns
Convolvulaceae	400	2	0.0006061	0.0179441	ns	0.0006016	0.0287148	ns
Costaceae	23	0	0.0000000	0.1481851	ns	0.0000000	0.3015404	ns
Coulaceae	1	0	0.0000000	0.9750000	ns	0.0000000	0.9936905	ns
Crassulaceae	1	0	0.0000000	0.9750000	ns	0.0000000	0.9936905	ns
Cucurbitaceae	146	2	0.0016633	0.0486066	ns	0.0016297	0.0765714	ns
Cunoniaceae	12	0	0.0000000	0.2646485	ns	0.0000000	0.4808911	ns
Cyclanthaceae	36	0	0.0000000	0.0973938	ns	0.0000000	0.2087019	ns
Cymodoceaceae	3	0	0.0000000	0.7075982	ns	0.0000000	0.8818828	ns
Cyperaceae	636	1	0.0000398	0.0087290	ns	0.0000396	0.0159494	ns
Cyrillaceae	1	0	0.0000000	0.9750000	ns	0.0000000	0.9936905	ns
Dichapetalaceae	26	0	0.0000000	0.1322746	ns	0.0000000	0.2735152	ns
Dilleniaceae	78	0	0.0000000	0.0461924	ns	0.0000000	0.1044437	ns
Dioscoreaceae	136	2	0.0017859	0.0521120	ns	0.0017473	0.0819470	ns
Droseraceae	32	0	0.0000000	0.1088812	ns	0.0000000	0.2305750	ns
Ebenaceae	62	2	0.0039308	0.1117191	ns	0.0037483	0.1704563	ns
Elaeocarpaceae	43	0	0.0000000	0.0822111	ns	0.0000000	0.1789644	ns
Elatinaceae	2	0	0.0000000	0.8418861	ns	0.0000000	0.9472550	ns
Ericaceae	106	1	0.0002388	0.0514431	ns	0.0002322	0.0912983	ns
Eriocaulaceae	591	0	0.0000000	0.0062223	Underused	0.0000000	0.0146882	ns
Erythropalaceae	22	0	0.0000000	0.1543725	ns	0.0000000	0.3121903	ns
Erythroxylaceae	133	0	0.0000000	0.0273548	ns	0.0000000	0.0631109	ns
Escalloniaceae	9	0	0.0000000	0.3362671	ns	0.0000000	0.5718585	ns
Euphorbiaceae	946	7	0.0029800	0.0151862	ns	0.0029706	0.0192930	ns
Euphroniaceae	1	0	0.0000000	0.9750000	ns	0.0000000	0.9936905	ns
Fabaceae	2857	21	0.0045556	0.0112140	ns	0.0045508	0.0124605	ns
Gelsemiaceae	2	0	0.0000000	0.8418861	ns	0.0000000	0.9472550	ns
Gentianaceae	124	0	0.0000000	0.0293109	ns	0.0000000	0.0674816	ns
Geraniaceae	7	0	0.0000000	0.4096164	ns	0.0000000	0.6524529	ns
Gesneriaceae	226	0	0.0000000	0.0161900	ns	0.0000000	0.0378056	ns
Goodeniaceae	1	0	0.0000000	0.9750000	ns	0.0000000	0.9936905	ns
Goupiaceae	1	0	0.0000000	0.9750000	ns	0.0000000	0.9936905	ns
Griseliniaceae	1	0	0.0000000	0.9750000	ns	0.0000000	0.9936905	ns
Gunneraceae	2	0	0.0000000	0.8418861	ns	0.0000000	0.9472550	ns
Haemodoraceae	5	0	0.0000000	0.5218238	ns	0.0000000	0.7551368	ns
Haloragaceae	5	0	0.0000000	0.5218238	ns	0.0000000	0.7551368	ns
Heliconiaceae	25	0	0.0000000	0.1371852	ns	0.0000000	0.2822644	ns
Hernandiaceae	11	0	0.0000000	0.2849142	ns	0.0000000	0.5079757	ns
Humiriaceae	37	1	0.0006840	0.1416031	ns	0.0006327	0.2366374	ns
Hydnoraceae	3	0	0.0000000	0.7075982	ns	0.0000000	0.8818828	ns
Hydrocharitaceae	13	0	0.0000000	0.2470526	ns	0.0000000	0.4564565	ns
Hydroleaceae	2	0	0.0000000	0.8418861	ns	0.0000000	0.9472550	ns
Hypericaceae	54	0	0.0000000	0.0660315	ns	0.0000000	0.1461991	ns
Hypoxidaceae	3	0	0.0000000	0.7075982	ns	0.0000000	0.8818828	ns
Icacinaceae	11	0	0.0000000	0.2849142	ns	0.0000000	0.5079757	ns
Iridaceae	198	0	0.0000000	0.0184582	ns	0.0000000	0.0429964	ns
Ixonanthaceae	4	0	0.0000000	0.6023646	ns	0.0000000	0.8159484	ns
Juncaceae	23	0	0.0000000	0.1481851	ns	0.0000000	0.3015404	ns
Juncaginaceae	2	0	0.0000000	0.8418861	ns	0.0000000	0.9472550	ns
Krameriaceae	5	0	0.0000000	0.5218238	ns	0.0000000	0.7551368	ns
Lacistemataceae	11	0	0.0000000	0.2849142	ns	0.0000000	0.5079757	ns
Lamiaceae	515	4	0.0021202	0.0197663	ns	0.0021079	0.0276439	ns
Lauraceae	461	0	0.0000000	0.0079700	ns	0.0000000	0.0187779	ns
Lecythidaceae	121	1	0.0002092	0.0451861	ns	0.0002042	0.0805345	ns
Lentibulariaceae	90	0	0.0000000	0.0401589	ns	0.0000000	0.0913878	ns
Lepidobotryaceae	1	0	0.0000000	0.9750000	ns	0.0000000	0.9936905	ns
Linaceae	15	0	0.0000000	0.2180194	ns	0.0000000	0.4141775	ns
Linderniaceae	12	0	0.0000000	0.2646485	ns	0.0000000	0.4808911	ns
Loasaceae	17	0	0.0000000	0.1950643	ns	0.0000000	0.3789268	ns
Loganiaceae	121	0	0.0000000	0.0300266	ns	0.0000000	0.0690761	ns
Loranthaceae	86	0	0.0000000	0.0419870	ns	0.0000000	0.0953616	ns
Lythraceae	222	0	0.0000000	0.0164793	ns	0.0000000	0.0384690	ns
Magnoliaceae	2	0	0.0000000	0.8418861	ns	0.0000000	0.9472550	ns
Malpighiaceae	581	3	0.0010661	0.0150152	ns	0.0010606	0.0222272	ns
Malvaceae	836	3	0.0007407	0.0104510	ns	0.0007380	0.0155001	ns
Marantaceae	220	1	0.0001151	0.0250641	ns	0.0001135	0.0452871	ns
Marcgraviaceae	34	0	0.0000000	0.1028179	ns	0.0000000	0.2190962	ns
Martyniaceae	3	0	0.0000000	0.7075982	ns	0.0000000	0.8818828	ns
Mayacaceae	4	0	0.0000000	0.6023646	ns	0.0000000	0.8159484	ns
Melastomataceae	1439	4	0.0007579	0.0071017	ns	0.0007563	0.0099761	ns
Meliaceae	92	1	0.0002752	0.0590779	ns	0.0002665	0.1043084	ns
Menispermaceae	108	1	0.0002344	0.0505105	ns	0.0002281	0.0896999	ns
Menyanthaceae	2	0	0.0000000	0.8418861	ns	0.0000000	0.9472550	ns
Metteniusaceae	16	0	0.0000000	0.2059072	ns	0.0000000	0.3957846	ns
Microteaceae	9	0	0.0000000	0.3362671	ns	0.0000000	0.5718585	ns
Molluginaceae	5	0	0.0000000	0.5218238	ns	0.0000000	0.7551368	ns
Monimiaceae	46	0	0.0000000	0.0770618	ns	0.0000000	0.1686589	ns
Moraceae	205	3	0.0030281	0.0421693	ns	0.0029843	0.0617255	ns
Muntingiaceae	1	0	0.0000000	0.9750000	ns	0.0000000	0.9936905	ns
Myristicaceae	64	1	0.0003955	0.0840103	ns	0.0003778	0.1458632	ns
Myrtaceae	1054	31	0.0200695	0.0414894	Overused	0.0200123	0.0446612	Overused
Nartheciaceae	2	0	0.0000000	0.8418861	ns	0.0000000	0.9472550	ns
Nyctaginaceae	61	0	0.0000000	0.0586812	ns	0.0000000	0.1309357	ns
Nymphaeaceae	23	1	0.0011002	0.2194866	ns	0.0009733	0.3486788	ns
Ochnaceae	207	0	0.0000000	0.0176628	ns	0.0000000	0.0411791	ns
Olacaceae	13	1	0.0019456	0.3602974	ns	0.0015811	0.5237708	ns
Oleaceae	14	0	0.0000000	0.2316358	ns	0.0000000	0.4343179	ns
Onagraceae	62	0	0.0000000	0.0577626	ns	0.0000000	0.1290113	ns
Opiliaceae	5	0	0.0000000	0.5218238	ns	0.0000000	0.7551368	ns
Orchidaceae	2340	0	0.0000000	0.0015752	Underused	0.0000000	0.0037373	Underused
Orobanchaceae	41	0	0.0000000	0.0860438	ns	0.0000000	0.1865620	ns
Oxalidaceae	108	0	0.0000000	0.0335796	ns	0.0000000	0.0769556	ns
Passifloraceae	164	10	0.0296245	0.1092759	Overused	0.0290853	0.1294375	Overused
Pentaphylacaceae	19	0	0.0000000	0.1764669	ns	0.0000000	0.3491221	ns
Peraceae	18	0	0.0000000	0.1853020	ns	0.0000000	0.3634240	ns
Peridiscaceae	1	0	0.0000000	0.9750000	ns	0.0000000	0.9936905	ns
Phyllanthaceae	133	0	0.0000000	0.0273548	ns	0.0000000	0.0631109	ns
Phytolaccaceae	11	0	0.0000000	0.2849142	ns	0.0000000	0.5079757	ns
Picramniaceae	22	0	0.0000000	0.1543725	ns	0.0000000	0.3121903	ns
Picrodendraceae	4	0	0.0000000	0.6023646	ns	0.0000000	0.8159484	ns
Piperaceae	462	2	0.0005247	0.0155497	ns	0.0005213	0.0249137	ns
Plantaginaceae	126	2	0.0019281	0.0561622	ns	0.0018831	0.0881340	ns
Plumbaginaceae	2	0	0.0000000	0.8418861	ns	0.0000000	0.9472550	ns
Poaceae	1297	2	0.0001868	0.0055591	Underused	0.0001864	0.0089526	ns
Polygalaceae	213	1	0.0001189	0.0258790	ns	0.0001172	0.0467335	ns
Polygonaceae	84	1	0.0003014	0.0645520	ns	0.0002910	0.1135534	ns
Pontederiaceae	26	0	0.0000000	0.1322746	ns	0.0000000	0.2735152	ns
Portulacaceae	20	1	0.0012651	0.2487328	ns	0.0011002	0.3878119	ns
Potamogetonaceae	13	0	0.0000000	0.2470526	ns	0.0000000	0.4564565	ns
Primulaceae	141	1	0.0001795	0.0388805	ns	0.0001758	0.0695934	ns
Proteaceae	37	0	0.0000000	0.0948906	ns	0.0000000	0.2038647	ns
Putranjivaceae	3	0	0.0000000	0.7075982	ns	0.0000000	0.8818828	ns
Quiinaceae	35	0	0.0000000	0.1000324	ns	0.0000000	0.2137733	ns
Quillajaceae	1	0	0.0000000	0.9750000	ns	0.0000000	0.9936905	ns
Ranunculaceae	15	0	0.0000000	0.2180194	ns	0.0000000	0.4141775	ns
Rapateaceae	41	0	0.0000000	0.0860438	ns	0.0000000	0.1865620	ns
Rhabdodendraceae	3	0	0.0000000	0.7075982	ns	0.0000000	0.8818828	ns
Rhamnaceae	44	5	0.0379437	0.2455768	Overused	0.0354563	0.3080913	Overused
Rhizophoraceae	10	0	0.0000000	0.3084971	ns	0.0000000	0.5381315	ns
Rosaceae	29	3	0.0218637	0.2735152	Overused	0.0197672	0.3643923	Overused
Rubiaceae	1	0	0.0000000	0.9750000	ns	0.0000000	0.9936905	ns
Ruppiaceae	194	3	0.0032005	0.0445247	ns	0.0031515	0.0651099	ns
Rutaceae	1388	4	0.0007857	0.0073621	ns	0.0007841	0.0103409	ns
Sabiaceae	9	0	0.0000000	0.3362671	ns	0.0000000	0.5718585	ns
Salicaceae	99	0	0.0000000	0.0365757	ns	0.0000000	0.0835533	ns
Samydaceae	3	0	0.0000000	0.7075982	ns	0.0000000	0.8818828	ns
Santalaceae	54	0	0.0000000	0.0660315	ns	0.0000000	0.1461991	ns
Sapindaceae	418	3	0.0014825	0.0208301	ns	0.0014719	0.0307607	ns
Sapotaceae	237	6	0.0093461	0.0542860	Overused	0.0092286	0.0699907	Overused
Sarraceniaceae	5	0	0.0000000	0.5218238	ns	0.0000000	0.7551368	ns
Schlegeliaceae	7	0	0.0000000	0.4096164	ns	0.0000000	0.6524529	ns
Schoepfiaceae	5	0	0.0000000	0.5218238	ns	0.0000000	0.7551368	ns
Scrophulariaceae	17	0	0.0000000	0.1950643	ns	0.0000000	0.3789268	ns
Simaroubaceae	37	0	0.0000000	0.0948906	ns	0.0000000	0.2038647	ns
Siparunaceae	20	0	0.0000000	0.1684335	ns	0.0000000	0.3358891	ns
Smilacaceae	32	0	0.0000000	0.1088812	ns	0.0000000	0.2305750	ns
Solanaceae	468	9	0.0088303	0.0361911	ns	0.0087738	0.0440812	ns
Staphyleaceae	1	0	0.0000000	0.9750000	ns	0.0000000	0.9936905	ns
Stemonuraceae	1	0	0.0000000	0.9750000	ns	0.0000000	0.9936905	ns
Strelitziaceae	1	0	0.0000000	0.9750000	ns	0.0000000	0.9936905	ns
Strombosiaceae	2	0	0.0000000	0.8418861	ns	0.0000000	0.9472550	ns
Styracaceae	25	0	0.0000000	0.1371852	ns	0.0000000	0.2822644	ns
Surianaceae	1	0	0.0000000	0.9750000	ns	0.0000000	0.9936905	ns
Symplocaceae	45	0	0.0000000	0.0787051	ns	0.0000000	0.1719599	ns
Taccaceae	1	0	0.0000000	0.9750000	ns	0.0000000	0.9936905	ns
Talinaceae	2	2	0.1581139	1.0000000	Overused	0.0527450	1.0000000	Overused
Tetrameristaceae	1	0	0.0000000	0.9750000	ns	0.0000000	0.9936905	ns
Theaceae	1	0	0.0000000	0.9750000	ns	0.0000000	0.9936905	ns
Thismiaceae	16	0	0.0000000	0.2059072	ns	0.0000000	0.3957846	ns
Thurniaceae	2	0	0.0000000	0.8418861	ns	0.0000000	0.9472550	ns
Thymelaeaceae	25	0	0.0000000	0.1371852	ns	0.0000000	0.2822644	ns
Tofieldiaceae	4	0	0.0000000	0.6023646	ns	0.0000000	0.8159484	ns
Trigoniaceae	26	0	0.0000000	0.1322746	ns	0.0000000	0.2735152	ns
Triuridaceae	13	0	0.0000000	0.2470526	ns	0.0000000	0.4564565	ns
Tropaeolaceae	4	0	0.0000000	0.6023646	ns	0.0000000	0.8159484	ns
Turneraceae	163	0	0.0000000	0.0223770	ns	0.0000000	0.0519043	ns
Typhaceae	3	2	0.0942993	0.9915962	Overused	0.0432719	0.9957893	Overused
Ulmaceae	6	0	0.0000000	0.4592581	ns	0.0000000	0.7007049	ns
Urticaceae	108	2	0.0022506	0.0652965	ns	0.0021896	0.1019933	ns
Velloziaceae	225	0	0.0000000	0.0162614	ns	0.0000000	0.0379693	ns
Verbenaceae	284	0	0.0000000	0.0129050	ns	0.0000000	0.0302424	ns
Violaceae	78	0	0.0000000	0.0461924	ns	0.0000000	0.1044437	ns
Vitaceae	50	1	0.0005062	0.1064695	ns	0.0004776	0.1821078	ns
Vivianiaceae	2	0	0.0000000	0.8418861	ns	0.0000000	0.9472550	ns
Vochysiaceae	166	0	0.0000000	0.0219771	ns	0.0000000	0.0509987	ns
Winteraceae	3	0	0.0000000	0.7075982	ns	0.0000000	0.8818828	ns
Ximeniaceae	5	0	0.0000000	0.5218238	ns	0.0000000	0.7551368	ns
Xyridaceae	198	0	0.0000000	0.0184582	ns	0.0000000	0.0429964	ns
Zingiberaceae	20	0	0.0000000	0.1684335	ns	0.0000000	0.3358891	ns
Zygophyllaceae	4	0	0.0000000	0.6023646	ns	0.0000000	0.8159484	ns
Total geral	32,740	254	0.0068357	0.0088651		0.0068357	0.0088651	

nj, number of species for group J; xj, number of food species of group J; lower (*B*), lower limit for the Bayesian model; upper (*B*), upper limit for the Bayesian model; status (*B*), status according to the Bayesian model; lower (*I*), lower limit for the IDM; upper (*I*), upper limit for the IDM; status (*I*), status according to the IDM

All overused and underused families found with the IDM approach were the same as those found with the Bayesian approach (Anacardiaceae, Annonaceae, Arecaceae, Cactaceae, Capparaceae, Caryocaraceae, Myrtaceae, Passifloraceae, Rhamnaceae, Rosaceae, Sapotaceae, Talinaceae, and Typhaceae were overused, and Orchidaceae was underused), since the latter was less conservative in relation to the IDM approach. Thus, in the Bayesian model, in addition to the families found with the IDM approach, there was one more family considered overused (Basellaceae) and two additional families considered underused (Eriocaulaceae and Poaceae).

## Discussion

The results found in this review provide further evidence supporting the theory of non-random selection of plants, in this case, of wild food plants in Brazil. Similar findings have been reported in different socioecological contexts for medicinal plants such as in Brazil [57], India [4], Papua New Guinea [3], Italy [58], Ecuador [59], Africa [6], Europe [60], Nepal [7], and South Africa [8].

The results of this study were consistent with those observed in the literature for medicinal plants. For example, in a study conducted in Brazil regarding medicinal plants, with a similar methodology to the one employed here (using Bayesian and IDM approaches), the families Anacardiaceae, Capparaceae, Caryocaraceae, Rhamnaceae, and Rosaceae were identified as overused, while Eriocaulaceae, Orchidaceae, and Poaceae were considered underused [57]. In Italy, a study using linear regression, the binomial method, and the Bayesian approach showed that Rosaceae was overused while Poaceae and Orchidaceae were underused [58], similar to the findings in our study. In Papua New Guinea, using the Bayesian approach, Anacardiaceae and Arecaceae were considered overused, and Poaceae and Orchidaceae underused [3]. In India, also with the Bayesian approach, Anacardiaceae and Cactaceae were found to be overused and with a binomial analysis, Poaceae showed to be underused [4]. Finally, in a review with useful plants from Chile, specifically those of the edible category, the families Myrtaceae, Cactaceae and Anacardiaceae were considered overused through the IDM and Bayesian approaches [5], similar to the results found in the present review.

It is worth noting that attractive factors differ between food and medicinal plants, especially from a physicochemical point of view. When similar results are found in the two categories, this does not necessarily mean that the same selection criteria apply for both. The fact that some families are concomitantly overused or underused in both categories may indicate that physicochemical properties are not the only aspect that leads a taxonomic group to be chosen or not. For example, Orchidaceae usually occurs at a low frequency in the environment and most of its plants grow as epiphytes; these characteristics could hinder experimentation in this group of plants and their consequent incorporation into medicinal and food systems.

Since the physicochemical requirements for the selection of medicinal and food plants differ, other shared factors are likely responsible for several families being overused for both purposes. The fact that some families are concomitantly under- or overexplored for food and medicinal purposes, as found in this review and in other phytosociological studies carried out in Brazil, may be related to the ease of access, because many species are widely dominant in Brazilian ecosystems. For example, Anacardiaceae was among the richest families in studies carried out in the Atlantic Forest with native species [61] and also in Caatinga, in an anthropized area [62]. Arecaceae was one of the species with the highest number of species in a study conducted in the Amazon [63]. Myrtaceae and Anacardiaceae were very well represented in terms of number of species in Cerrado [64]. The good representativity of species of these families in the environment is likely a contributing factor for people to find them easily, leading to more contact and greater chances of identifying their uses, ultimately causing these families to stand out as families of both medicinal and food plants.

Besides the ease of access, it is possible that these plants have other attractive characteristics. For example, various studies carried out in Brazil have identified the fruit of food species as the most used plant organ [27, 43, 44, 65]. The absence of such attractive characteristics may explain why some underutilized families have few or none species mentioned as food plant in the wild group, such as Orchidaceae, Eriocaulaceae, and Poaceae in this review. In the case of the latter, despite the family has representatives of great economic importance worldwide and this could theoretically encourage the use of other species of the family, this did not happen in the present review. Only two out of a total of 1297 species of Poaceae from the native flora of Brazil were mentioned as wild food plants.

The results found in the literature indicate that families that have fleshy fruits, such as Arecaceae, Myrtaceae, and Passifloraceae, tend to be better known and used. Fruits of Myrtaceae are known to have a large number and concentration of phenolic compounds with important antioxidant properties, which are beneficial to human health [66]. Some fruits of the family Arecaceae have high nutritional value and are rich in bioactive compounds [67]. Passifloraceae fruits are rich in magnesium and zinc, in addition to containing phenolic compounds, triterpenes, steroids, and flavonoids [68]. These characteristics are key for the determination of their uses, because their presence can contribute to people selecting the plants for consumption.

## Conclusions

The selection of wild food plants occurring in Brazil, known and used by different populations, presents a marked taxonomic bias. The identification of overused and underused families contributes to the discovery of families with potential for popularization. In addition, this work is important from the point of view of conservation of wild plants and for the promotion of food and nutritional security. Therefore, efforts are needed to identify the species that could be incorporated into the diet of populations in view of characteristics that make plants more used in relation to others. Furthermore, investigating which parts are most used, their nutritional value, which are the forms of consumption, which are the promising species in the group of wild food species in Brazil, and defining strategies for the management of use are also fields yet to be explored.

In view of their wide geographical distribution, families such as Anacardiaceae Myrtaceae, Arecaceae, and Passifloraceae can be strategic for food prospecting aimed at popularization.

## Data Availability

The datasets generated and/or analyzed during the current study are available in the repository [Google Drive] [https://docs.google.com/spreadsheets/d/1hLIJT54-r0-06Obg-RGCIN7yS4uOf4i9/edit#gid=1936773480]. The datasets generated during and/or analyzed during the current study are available from the corresponding author on reasonable request.
